# Lipoprotein particle sizes and incident type 2 diabetes: the PREVEND cohort study

**DOI:** 10.1007/s00125-021-05603-3

**Published:** 2021-11-20

**Authors:** Sara Sokooti, José L. Flores-Guerrero, Hiddo J. L. Heerspink, Erwin Garcia, Margery A. Connelly, Stephan J. L. Bakker, Robin P. F. Dullaart

**Affiliations:** 1 Department of Internal Medicine, University Medical Center Groningen, University of Groningen, Groningen, the Netherlands; 2 Department of Clinical Pharmacy and Pharmacology, University Medical Center Groningen, University of Groningen, Groningen, the Netherlands; 3 Laboratory Corporation of America® Holdings (Labcorp), Morrisville, NC USA

**Keywords:** HDL size, LDL size, Risk prediction, TRL size, Type 2 diabetes

*To the Editor*: With much interest we read the paper by Carvalho et al, which demonstrated that including triacylglycerol-rich lipoproteins (TRL) particle diameter in a risk prediction model improved the risk prediction of incident type 2 diabetes after a mean 3.7 years of follow-up in the Estudo Longitudinal de Saúde do Adulto (Brazilian Longitudinal Study of Adult Health; ELSA-Brasil) cohort, particularly in participants with lower HbA_1c_ levels [1]. TRL particle diameter is a lipoprotein metric that is likely to undergo changes early in the course of the pathophysiological processes that lead to the development of type 2 diabetes, even before plasma glucose abnormalities are established [2].

We have reported that, in the Prevention of Renal and Vascular End-Stage Disease (PREVEND) cohort, TRL size was positively associated, whereas LDL size was inversely associated, with incident type 2 diabetes [3]. Notably, in the same population, we also found that HDL particle size and the concentration of large HDL particles were both inversely associated with incident type 2 diabetes [4], consistent with a protective effect of HDL on beta cell function [5]. Hence, it is plausible that including lipoprotein diameter markers (such as LDL and HDL size) other than TRL size alone in risk prediction models may improve the prediction of incident type 2 diabetes.

Here, we present novel data from our recent studies [3, 4], aiming to investigate whether, and to what extent, the size of TRL, LDL and HDL particles, and the ratios and combinations of these lipoprotein particle sizes, are independently associated with incident type 2 diabetes. Furthermore, we question whether the addition of LDL and/or HDL size to risk prediction models improves type 2 diabetes risk prediction over the inclusion of TRL size alone.

After exclusion of participants with missing data, a total of 4818 participants of the PREVEND study without type 2 diabetes at baseline were included in this study [3, 4]. After a median follow-up of 7.3 years, 263 participants had developed type 2 diabetes, which was determined if one or more of the following criteria were met: (1) fasting plasma glucose (FPG) ≥7.0 mmol/l; (2) random sample plasma glucose ≥11.1 mmol/l; (3) self-report of a physician diagnosis of type 2 diabetes; and (4) initiation of glucose-lowering medication use, retrieved from a central pharmacy registry. The protocol for the PREVEND study was approved by the local ethics committee of the University Medical Center Groningen (approval number: MEC96/01/022).

After an overnight fast, EDTA anti-coagulated plasma samples were obtained, and lipoprotein variables were measured by NMR LipoProfile testing at Labcorp (Morrisville, NC, USA). Lipoprotein variables were reported using an optimised version of the NMR LipoProfile test (LP4 algorithm) [3, 4, 6]. Mean TRL, LDL and HDL sizes were calculated using the weighted mean derived from the sum of the diameters of each subfraction. This contrasts with the ELSA-Brasil report, which relied on the earlier LP3 algorithm employed by the same laboratory; one important difference is that intermediate density lipoproteins are categorised as an LDL subfraction with the LP3 algorithm and as a VLDL subfraction with the LP4 algorithm. In view of the reciprocal relationship between TRL and HDL sizes, as well as between TRL and LDL sizes, lipoprotein particle size ratios were calculated by dividing the size of one lipoprotein fraction by the size of another lipoprotein fraction. We also calculated the combined TRL and HDL size variable, which was estimated by the combined predictive probability of TRL and HDL size related to incident type 2 diabetes using logistic regression analyses.

Crude and multivariable Cox proportional hazards regression analyses were performed to examine the associations between each lipoprotein particle size variable and incident type 2 diabetes. We estimated changes in the risk of developing diabetes with inclusion of TRL, LDL and HDL size, their ratios, and the combined TRL and HDL size variable using the Framingham Offspring (FOS) risk score, a traditional type 2 diabetes risk assessment tool that takes into account age, sex, family history of type 2 diabetes, BMI, BP and FPG, as a base model [7]. Harrell’s C-statistics were calculated with and without the inclusion of each lipoprotein particle size variable in the models, and differences were tested. Additionally, improvement of diabetes prediction was examined in terms of the net reclassification index (NRI) and integrated discrimination improvement (IDI) [8].

Higher values of TRL size, as well as higher ratios of TRL/LDL size, TRL/HDLsize, and LDL/HDL size, and the combined TRL and HDL size estimate, were associated with increased risk of type 2 diabetes, whereas both LDL and HDL size were associated with a lower risk of type 2 diabetes in the crude analysis (Table 1). Except for the LDL/HDL size ratio, the associations remained significant for all of the lipid variables in multivariable-adjusted Cox regression models after adjustment for multiple type 2 diabetes risk factors, including high alcohol intake (>3 units/day), BMI, lipid-lowering medication, anti-hypertensive medication, family history of diabetes, FPG and fasting plasma insulin. The association of TRL size with incident type 2 diabetes was similar to that of the TRL/HDL and the TRL/LDL size ratios, and the combined TRL and HDL size estimate (Table 1). Harrell’s C-index for the FOS risk score amounted to 0.879, which was not significantly improved with the addition of TRL size, LDL size or HDL size separately but was modestly increased to 0.884 with the addition of TRL/HDL size ratio, and to 0.883 with the addition of combined TRL and HDL size estimate (Table 2). Except for the LDL/HDL size ratio, IDI and NRI estimates were greater after the addition of each of the lipoprotein size indices to the FOS risk score (Table 2).

**Table 1:** Association between lipoprotein size variables and risk of type 2 diabetes in 4818 participants of the PREVEND cohort

	HR (95% CI) per SD increase						
Variable	TRL size	LDL size	HDL size	TRL/LDL size	TRL/HDL size	LDL/HDL size	Combined TRL and HDL size
Crude	1.68 (1.53–1.84)***	0.62 (0.57–0.69)***	0.50 (0.44–0.58)***	1.69 (1.54–1.85)***	1.76 (1.61–1.93)***	1.44 (1.26–1.63)***	1.61 (1.47–1.77)***
Model 1	1.65 (1.50–1.82)***	0.64 (0.58–0.71)***	0.48 (0.41–0.56)***	1.66 (1.52–1.83)***	1.74 (1.58–1.91)***	1.42 (1.23–1.63)***	1.59 (1.45–1.75)***
Model 2	1.45 (1.30–1.61)***	0.75 (0.67–0.84)***	0.60 (0.50–0.71)***	1.45 (1.31–1.61)***	1.51 (1.36–1.68)***	1.24 (1.07–1.44)**	1.41 (1.27–1.56)***
Model 3	1.32 (1.19–1.47)***	0.77 (0.69–0.87)***	0.64 (0.54–0.77)***	1.33 (1.20–1.48)***	1.37 (1.23–1.53)***	1.17 (1.01–1.35)*	1.29 (1.16–1.43)***
Model 4	1.26 (1.12–1.41)***	0.80 (0.71–0.90)***	0.69 (0.58–0.83)***	1.27 (1.14–1.43)***	1.31 (1.16–1.47)***	1.11 (0.96–1.29)	1.23 (1.10–1.38)***

HR (95% CI) for risk of type 2 diabetes, derived from Cox proportional hazard models and expressed per 1 SD increase in variables

Model 1, adjusted for sex and age; Model 2, model 1 + high alcohol intake (>3 units/day), BMI, lipid-lowering medication, anti-hypertensive medication and family history of diabetes; Model 3, model 2 + FPG; Model 4, model 3 + fasting plasma insulin

*p < 0.05; **p < 0.01; ***p < 0.001

**Table 2:** Additive value of lipoprotein size for the prediction of type 2 diabetes in 4818 participants of the PREVEND cohort

Model	C-statistics	*p* value for change in C-statistics	IDI	*p* value	NRI	*p* value
Base model	0.879 (0.859–0.899)	–	–	–	–	–
+TRL size	0.883 (0.863–0.903)	0.067	0.0119	0.0012	0.266	<0.001
+LDL size	0.881 (0.860–0.902)	0.587	0.0102	0.0011	0.254	<0.001
+HDL size	0.883 (0.864–0.903)	0.170	0.0064	0.0103	0.295	<0.001
+TRL/LDL size	0.883 (0.863–0.903)	0.071	0.0132	<0.001	0.292	<0.001
+TRL/HDL Size	0.884 (0.864–0.904)	0.041	0.0142	<0.001	0.327	<0.001
+LDL/HDL size	0.881(0.861–0.901)	0.149	−0.0005	0.6911	0.195	0.001
+Combined TRL and HDL size	0.883 (0.864–0.904)	0.031	0.0147	<0.001	0.380	<0.001

Base model defined as FOS risk score including age, sex, BMI, family history of diabetes, systolic BP, diastolic BP and FPG

In summary, these findings in a large population-based cohort corroborate the findings of Carvalho et al about the association between TRL size and incident type 2 diabetes [1]. However, we propose that although TRL, LDL and HDL size are all associated with incident type 2 diabetes after adjustment for relevant potential confounders, the TRL/HDL size ratio, and combined TRL and HDL size estimate seem more robust in improving type 2 diabetes risk prediction. Hence, reporting all major lipoprotein fractions is likely to be useful for the risk prediction of type 2 diabetes development.

## Data Availability

The datasets used and analysed during the current study are available from the corresponding author on reasonable request.

## Abbreviations

ELSA-Brasil: Estudo Longitudinal de Saúde do Adulto (Brazilian Longitudinal Study of Adult Health)
FOS: Framingham Offspring
FPG: Fasting plasma glucose
IDI: Integrated discrimination improvement
NRI: Net reclassification index
PREVEND: Prevention of Renal and Vascular End-stage Disease
TRL: Triacylglycerol-rich lipoproteins
